# Prevalence and Predictors of Adolescent Alcohol Use and Binge Drinking in the United States

**DOI:** 10.35946/arcr.v35.2.10

**Published:** 2014

**Authors:** Megan E. Patrick, John E. Schulenberg

**Affiliations:** **Megan E. Patrick, Ph.D.**, *is a research assistant professor at the Institute for Social Research, and*; **John E. Schulenberg, Ph.D.**, *is professor in the Department of Psychology and research professor at the Institute for Social Research, University of Michigan, Ann Arbor, Michigan.*

**Keywords:** Underage drinking, binge drinking, adolescent, high school student, young adult, prevalence, predictors, causes of alcohol and other drug use, risk factors, school risk factors, environmental risk factors, family risk factors, peer risk factors, gender differences, racial/ethnic differences, Monitoring the Future (MTF) Study, United States

## Abstract

Because alcohol use typically is initiated during adolescence and young adulthood and may have long-term consequences, the Monitoring the Future (MTF) study annually assesses various measures of alcohol use among 8th-, 10th-, and 12th-grade students. These analyses have found that although alcohol use among these age groups overall has been declining since 1975, levels remain high. Thus, in 2011 about one-quarter of 8th graders, one-half of 10th graders, and almost two-thirds of 12th graders reported drinking alcohol in the month preceding the interview. Binge drinking (i.e., consumption of five or more drinks in a row) was also prevalent. Specific rates of drinking, binge drinking, and getting drunk varied among different student subgroups based on gender and race/ethnicity. The MTF study has also identified numerous factors that influence the risk of alcohol use among adolescents, including parents and peers, school and work, religiosity and community attachment, exercise and sports participation, externalizing behavior and other drug use, risk taking and sensation seeking, well-being, and drinking attitudes and reasons for alcohol use. Drinking during adolescence can have long-term effects on a person’s life trajectory. Therefore, these findings have broad implications for prevention and intervention efforts with this population.

In the United States, alcohol use typically begins and escalates during adolescence and young adulthood. To describe the historical and developmental trends in substance use in this age group, the Monitoring the Future (MTF) study (Johnston et al. 2012) was designed in 1975. Since then, this ongoing national-cohort sequential longitudinal study assessing the epidemiology and etiology of substance use among adolescents and adults has been funded by the National Institute on Drug Abuse (NIDA). This article summarizes findings from the MTF study regarding the prevalence and predictors of alcohol use during adolescence.

## The Prevalence of Drinking and Historical Changes

As is true for adults, alcohol is the most commonly abused substance among American youth. The MTF study has been documenting the prevalence and trends in alcohol use frequency and binge drinking (i.e., consumption of five or more drinks in a row) for the past several decades in annual, national samples of American 8th-, 10th-, and 12th-grade students. Using these data, Patrick and Schulenberg (2010) found that very few 8th- and 10th-grade students who reported having ever used alcohol had not used alcohol in the past year, suggesting that most of the alcohol use reported is relatively recent. Therefore, this article focuses on alcohol use in the past 12 months and the past 30 days, as well as self-reported drunkenness in the past 30 days and binge drinking in the past 2 weeks. The prevalence figures for these variables for 2011 are summarized in table 1, broken down by grade level, gender, and racial/ethnic subgroups (for more information, see Johnston et al. 2012).

In 2011, 27 percent of 8th graders, 50 percent of 10th graders, and 64 percent of 12th graders reported having used alcohol in the past 12 months. The corresponding rates for alcohol use in the past 30 days were 13 percent, 27 percent, and 40 percent, respectively. Furthermore, 4 percent of 8th graders, 14 percent of 10th graders, and 25 percent of 12th graders reported having been drunk within the past month. Finally, binge drinking in the past 2 weeks was reported by 6 percent of 8th graders, 15 percent of 10th graders, and 22 percent of 12th graders.

Interestingly, it is more common for students to report binge drinking 2 or more times in the past 2 weeks than to report binge drinking only once in the past 2 weeks; thus, 61 percent of 8th graders and 62 percent of 10th graders who had engaged in binge drinking in the previous 2 weeks did so on multiple occasions (Patrick and Schulenberg 2010). This observation suggests a fast shift to frequent heavier drinking for many young people. In addition, the surveys indicate that extreme binge drinking (i.e., consumption of 10 or more or 15 or more drinks in a row) is a problem among 12th graders (this variable was not assessed among 8th and 10th graders). Thus, 10.5 percent of high school seniors reported consuming 10 or more drinks in a row, and 5.6 percent reported consuming 15 or more drinks in a row in the past 2 weeks (Patrick et al. 2013).

Alcohol use differs not only by age but also by demographic subgroups, including gender and race/ethnicity (see table 1). In 8th grade, girls tend to have somewhat higher rates of alcohol use (i.e., 13 percent in the past 30 days) than do boys (12 percent). Among older students, however, this ratio is reversed, with 38 percent of female and 42 percent of male 12th graders reporting alcohol use in the past 30 days. This gender difference continues into adulthood, with men consistently using alcohol at higher rates compared with women (Johnston et al. 2012; Wilsnack et al. 2000). A similar interaction seems to exist between grade and race/ethnicity (Wallace et al. 2003). Thus, among 8th graders, Hispanic youth tend to report a greater prevalence of alcohol consumption in the last 12 months (36 percent) or last 30 days (18 percent), as well as of being drunk in the last 30 days (6 percent) and binge drinking in the past 2 weeks (10 percent) than do both White and African American youth. By 12th grade, however, White adolescents have the highest prevalence levels of the three racial/ethnic groups on all alcohol use measures, African American adolescents have the lowest levels, and Hispanics have intermediate levels. For example, for binge-drinking, prevalence rates among 12th graders are 26 percent for Whites, 11 percent for African Americans, and 21 percent for Hispanics. Some, but not all, of these race/ethnicity differences in alcohol use among 12th graders are attributable to differential high-school dropout rates among the different groups. Thus, dropout rates tend to be higher among racial/ethnic minority youth, and alcohol and other drug (AOD) use tends to be higher among school dropouts than among those staying in school (Bachman et al. 2008).

Overall, alcohol use among adolescents and young adults has been declining to the lowest levels in recent decades, as shown by the trends in self-reported alcohol use in the past 12 months and drunkenness in the past 30 days (see figure 1) (Johnston et al. 2012; Patrick and Schulenberg 2010). Similar trends have been observed for alcohol use in the past 30 days and binge drinking in the past 2 weeks. These historical shifts in AOD use can be attributed to multiple influences. For example, changes in the minimum legal drinking age (e.g., Wagenaar et al. 2001) as well as in perceived social norms (e.g., Keyes et al. 2012) have been shown to contribute to changes in alcohol use. Of particular interest are historical shifts that relate to changes in developmental trajectories. Latent growth modeling analyses with multicohort data have demonstrated that, compared with earlier cohorts, more recent cohorts exhibit lower initial levels of binge drinking but more rapid increases from age 18 to young adulthood (Jager et al. 2013). This acceleration of alcohol use helps explain the findings that use among adolescents has been decreasing at faster rates than among young adults in recent decades.

## Predictors of Alcohol Use Among Adolescents

Despite the changes in alcohol use that have occurred over the past three decades, the relevant risk and protective factors tend to remain very stable across historic time, age, gender, and race/ethnicity (e.g., Brown et al. 2001; Donovan et al. 1999; Patrick and Schulenberg 2010). Like many other large-scale studies on adolescent AOD use, the MTF study has cast a wide net in terms of risk and protective factors, correlates, and consequences of substance use. Not only is this approach well suited to placing alcohol use within the larger context of adolescent development, it makes good use of the MTF large-scale survey approach that emphasizes breadth of measurement. Conceptually, the analyses drew from broad multidomain models when examining causes, correlates, and outcomes of adolescent alcohol use (e.g., Brown et al. 2009; Chassin et al. 2009; Maggs and Schulenberg 2005). This section summarizes MTF study findings concerning several domains of predictors of AOD use during adolescence, after considering methodological issues when examining causes and consequences of adolescent alcohol use.

### Methodological Issues in Understanding Risk Factors for and Consequences of Adolescent Alcohol Use

When considering the correlates of AOD use, any attempt to discern whether these correlates are causes or consequences of substance use is hampered by three factors:

- Firm conclusions about causal connections are difficult without randomly controlled experiments.
- Alcohol use during adolescence typically is reciprocally related to risk factors across development, such that problems that contribute to alcohol use may get worse with continued alcohol use (e.g., Cairns and Cairns, 1994; Dodge et al. 2009; Schulenberg and Maslowsky 2009).
- Factors that are identified as causes or as consequences of alcohol use during adolescence in the total sample likely do not apply to all young people, given the heterogeneity in developmental course (Schulenberg 2006).

Cross-sectional studies, in which each individual is evaluated only once, typically provide little leverage for concluding whether a given construct is a cause, correlate, or consequence of alcohol use, emphasizing the importance of conceptual guidance, logic, and statistical controls. Furthermore, when adolescents report using multiple substances, it is difficult to determine whether they are using the drugs simultaneously or whether use of one substance leads to use of another. Longitudinal panel studies, in which the same individuals are followed over time, provide more leverage but still leave room for alternative interpretations. For example, these studies may suffer from selection effects—that is, a construct excluded from the analysis actually “causes” both drug use and assumed consequence of drug use, rendering the relationship between cause and consequence spurious. Some recent analytic strategies that have been used with longitudinal data, such as propensity score analyses (Bachman et al. 2011) and fixed effects analysis (Patrick et al. 2012*a*; Staff et al. 2009), allow for greater control of selection effects and thus better leverage on likely causal connections. Nevertheless, despite such statistical advances, experiments in which participants are randomly assigned to experimental groups remain the gold standard for demonstrating causal connections.

Finally, the use of self-report data may limit the usefulness of study findings because such data rely on participants to remember and accurately perceive their own level of substance use. Nevertheless, most studies like the MTF study rely on these measures, because they have been found to be valid and reliable (Bachman et al. 2011; O’Malley et al. 1983) and because it is very expensive and burdensome to collect physiological data (e.g., blood, urine, or hair) and/or information from multiple reporters (e.g., parents or peers) in large-scale studies.

### Influence of Parents and Peers

One developmental transition characteristic of adolescence is the movement away from parents and increasing involvement with peers. Nonetheless, parents still play a pivotal role in adolescent experiences and in fact can sometimes counter the effects of other risk factors for AOD use. Like many other reports in the literature (e.g., Dishion and McMahon 1998; Kiesner et al. 2009), the MTF study found that parental supervision and monitoring relate to lower AOD use among 8th and 10th graders and together are one of the strongest predictors (Dever et al. 2012; Pilgrim et al. 2006). Of particular importance, this effect was equally important (i.e., invariant) across gender and race/ethnicity (Pilgrim et al. 2006). Furthermore, parental monitoring was especially protective against substance use for high-risk–taking adolescents (Dever et al. 2012).

The literature for decades has indicated that peer use is one of the strongest correlates of AOD use. This was confirmed in the MTF; thus, in an analysis of multiple predictors of binge drinking among 8th and 10th graders from 1991 to 2007, having friends who get drunk was the strongest risk factor, regardless of the grade level or cohort analyzed (Patrick and Schulenberg 2010). Moreover, friends’ alcohol use in high school predicted both concurrent binge drinking and future trajectories of binge drinking (Schulenberg et al. 1996). Overall, the frequency of evenings out with friends (unsupervised by adults) was associated with more AOD use (Bachman et al. 2008; Brown et al. 2001; Patrick and Schulenberg 2010). Of course, a central issue when evaluating the role of peer use as a correlate and predictor of alcohol use is the extent to which friends actually influence an individual or the individual select friends who, like them, already drink. During adolescence and the transition to adulthood, both of these processes typically play a role (e.g., Patrick et al. 2012*b*).

### Influence of School and Work

The broad domain of education also significantly relates to AOD use during adolescence (Crosnoe 2011). Studies consistently have found that grades, educational expectations, and school bonding are negatively correlated with AOD use, whereas school disengagement, school failure, school misbehavior, and skipping school are positively correlated with AOD use (Bachman et al. 2008; Bryant et al. 2003; Dever et al. 2012; Patrick and Schulenberg 2010; Pilgrim et al. 2006; Schulenberg et al. 1994). For example, in a longitudinal analysis examining 8th-grade predictors of concurrent and subsequent AOD use, school misbehavior and peer encouragement of misbehavior were positively associated with concurrent substance use and increased substance use over time. Conversely, school bonding, interest, and effort were negatively associated with concurrent and increased substance use, as were academic achievement and parental help with school (Bryant et al. 2003). Positive school attitudes were of particular importance and were especially influential as protective factors against substance use for low-achieving adolescents. The relationship between educational factors and AOD use is bidirectional, and it is clear that AOD use can contribute to educational difficulties. In general, however, it seems that based on MTF study longitudinal data and careful consideration of selection factors, the more common direction of influence is that school difficulties contribute to AOD use during adolescence (Bachman et al. 2008).

By the time they leave high school, most adolescents have worked part time during the school year. Although it has long been recognized that hours of work during adolescence are positively related to use of AODs, conclusions about causal connections have remained elusive (Staff et al. 2009). Analysis of MTF study data found that when sociodemographic and educational characteristics are controlled for, the positive relationship between hours of work and AOD use diminishes, suggesting that selection effects exist. In other words, long hours of work and substance use have a common set of causes, particularly disengagement from school (Bachman and Schulenberg 1993; Bachman et al. 2011). The influence of selection effects is further supported by findings that simply wanting to work long hours is associated with heavier AOD use. This is true regardless of actual hours spent working, and especially among those who do not work (Bachman et al. 2003; Staff et al. 2010).

### Religiosity and Community Attachment

Numerous studies found that religiosity tends to be negatively correlated with AOD use during adolescence (Brown et al. 2001; Wallace et al. 2003, 2007; Wray-Lake et al. 2012). This is true for both African American and White youth. In fact, religiosity does not explain race differences in substance use (Wallace et al. 2003). Religiosity tends to operate at both the individual and contextual levels, because highly religious adolescents attending highly religious schools have lower alcohol use compared with highly religious adolescents attending non–highly religious schools (Wallace et al. 2007). More broadly, community attachments, including religiosity as well as social trust and social responsibility, tend to be negatively correlated with AOD use during adolescence (Wray-Lake et al. 2012).

### Exercise and Sports Participation

Whereas exercise correlates negatively with alcohol use, participating in team sports correlates positively with alcohol use during high school (Terry-McElrath et al. 2011). This is especially true for males (Dever et al. 2012).

### Externalizing Behaviors and Other Drug Use

As part of a broader set of problem behaviors, it is not surprising that alcohol use is associated with externalizing behaviors as well as cigarette smoking and illicit drug use during adolescence. In the MTF study, externalizing behaviors overall, and aggressive behavior and theft/property damage in particular, correlated with AOD use during adolescence (Bachman et al. 2008; Brown et al. 2001; Maslowsky and Schulenberg, in press; Patrick and Schulenberg 2010). Disentangling causal connections is difficult, however, and it is likely that alcohol use both contributes to and is caused by externalizing behaviors (Osgood et al. 1988), particularly if these behaviors involve spending unsupervised time with peers (Osgood et al. 1996). Cigarette smoking and other illicit drug use also tend to be highly correlated with alcohol use during adolescence (Patrick and Schulenberg 2010).

### Risk Taking and Sensation Seeking

The willingness to take risks and high levels of sensation seeking also both correlate with higher levels of AOD use (Dever et al. 2012; Patrick and Schulenberg 2010; Pilgrim et al. 2006; Schulenberg et al. 1996). Among 8th graders and 10th graders, the impact of risk taking on substance use (including alcohol) was partly mediated through school bonding (which negatively affected AOD use) and time with friends (which positively affected AOD use); these effects were largely invariant across race/ethnicity and gender (Pilgrim et al. 2006).

### Well-Being

Self-esteem tends to be negatively correlated with AOD use and, correspondingly, self-derogation and depressive affect tend to be positively correlated with AOD use during adolescence (Maslowsky and Schulenberg, in press; Patrick and Schulenberg 2010; Schulenberg et al. 1996). When examining the relative contributions of conduct problems, depressive affect, and the interaction of conduct problems and depressive affect on AOD use, depressive affect is not as powerful a predictor as are conduct problems. However, the interaction of the two variables (i.e., high levels of both) is a relatively powerful predictor of alcohol use, especially for younger adolescents (Maslowsky and Schulenberg, in press).

### Drinking Attitudes and Reasons for Using Alcohol

Attitudes regarding alcohol use and reasons for use are powerful correlates and predictors of drinking behavior. Indeed, disapproval of binge drinking is one of the strongest protective factors against heavy drinking (Patrick and Schulenberg 2010). A long-standing focus of the MTF study has been to show how, at the population level, changes in perceptions of risk about and disapproval of substance use precede changes in substance use (Bachman et al. 1998; Johnston et al. 2012; Keyes et al. 2011). A recent analysis assessed the effects of age, period (i.e., the year in which data were obtained), and cohort effects of population-based social norms regarding heavy alcohol use (i.e., level of disapproval of heavy use) on individual-level heavy drinking during adolescence. The study found that cohort effects predominated, indicating that being part of a birth cohort that reported higher disapproval of heavy drinking set the stage for lower alcohol use (Keyes et al. 2012).

Motivations or reasons for drinking also are associated with alcohol use behaviors and may serve as a marker for the development of problematic behavioral patterns. The reasons for alcohol use typically change across adolescence and into adulthood. MTF study investigators have assessed reasons for drinking using MTF study panel data following high-school seniors into young adulthood. (MTF survey questions regarding motivations are not included in the 8th- and 10th-grade surveys.) Of particular interest here, 12th-grade adolescents tend to report higher motivation for drinking to get drunk (as well as other social and coping reasons for drinking) than do young adults. Conversely, 12th graders report lower motivations to use alcohol to relax, to sleep, and because it tastes good, all of which increase across the transition to adulthood (Patrick and Schulenberg 2011; Patrick et al. 2011). It is important to understand the reasons for alcohol use among adolescents, because the reasons for use reported in 12th grade, when adolescents are about 18, show long-term longitudinal associations with alcohol use and symptoms of alcohol use disorders decades later (Patrick et al. 2011; Schulenberg et al. 1996).

## Long-Term Consequences of Alcohol Use

Attempting to discern long-term consequences of adolescent AOD use is fraught with conceptual and methodological complexities (e.g., Schulenberg et al. 2003), yet it is critical for understanding the development (i.e., etiology) of adult alcohol use disorders. Numerous studies have demonstrated that alcohol use in middle school and high school may be an important indicator of later problems. For example, although most students mature out of their heavy alcohol use (Schulenberg and Maggs 2002; Schulenberg and Patrick 2012; Schulenberg et al. 1996), substance use in high school is one of the strongest predictors of substance use in adulthood. Specifically, binge drinking in 12th grade predicts symptoms of alcohol use disorders 17 years later, at age 35 (Merline et al. 2004, 2008; Patrick et al. 2011). Furthermore, trajectories of binge drinking are predictive of alcohol use disorders during middle adulthood (Schulenberg and Patrick 2012), and continued substance use into young adulthood is associated with HIV-related risk behaviors (Patrick et al. 2012). Finally, binge drinking in high school predicts subsequent dropping out of college, although an increase in binge drinking during college is related to not dropping out (Schulenberg and Patrick 2012).

## Implications for Prevention and Intervention

Studies on the etiology and epidemiology of alcohol use ought to go hand in hand in order to combine the broader approach of epidemiology with the more in-depth emphasis of etiology. As the discussion in this article has shown, there are both historical and developmental predictors related to adolescent AOD use that are changing over time. Understanding the scope of alcohol use during the middle-school and high-school years, and associated long-term problems, is an important step toward effectively intervening to reduce high-risk drinking and its negative consequences. The scope of the problem is underscored by the findings that more than one in five American high-school seniors in the class of 2011 reported binge drinking in the previous 2 weeks. The documented developmental increases in alcohol use across adolescence and young adulthood make this a particularly important time for intervention. In particular, the fast escalation among adolescents from binge drinking once to binge drinking multiple times within a given 2-week period (Patrick and Schulenberg 2010) highlights the importance of preventing early initiation as well as early escalation of AOD use.

Levels of alcohol use have been declining in recent decades, suggesting that past interventions, such as increasing the minimum legal drinking age to 21, have been effective. However, although it is worth recognizing that most adolescents manage to avoid heavy alcohol use and that such use is not an inevitable developmental progression, alcohol remains the most commonly used substance among adolescents, and its use is a leading cause of death and injury (U.S. Department of Health and Human Services 2007). To design effective programs and target prevention efforts toward students most likely to develop problematic levels of alcohol use, it is essential to identify characteristics of individuals at greatest risk. This effort is aided by the fact that the importance of risk and protective factors tends to remain very stable over time. As summarized above, demographic differences in drinking behavior point to important subgroups that should be targeted, including young men and White and Hispanic adolescents. Finally, the findings described here point to several risk and protective factors to consider when designing prevention and intervention programs, including parental involvement, peer influences, academic success, religiosity, externalizing and internalizing behaviors, alcohol attitudes, and self-reported reasons for drinking.

## Figures and Tables

**Figure:**
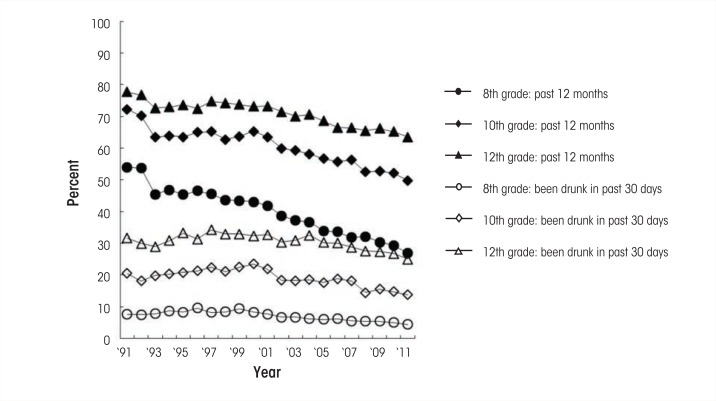
Trends in alcohol use in the past 12 months and in having been drunk in the past 30 days for 8th, 10th, and 12th graders, 1991–2011.

**Table 1:** Prevalence of Alcohol Use (%) by Demographic Subgroups in 8th, 10th, and 12th Graders, 2011

	**Any Use in Past 12 Months**	**Any Use in Past 30 Days**	**Been Drunk in Past 30 Days**	**5+ Drinks in a Row in Past 2 Weeks**
**8th Graders**				
Total	26.9	12.7	4.4	6.4
Gender				
Boys	26.2	12.1	4.4	6.1
Girls	27.1	12.8	4.2	6.5
Race/Ethnicity*				
White	26.2	12.3	4.7	6.2
African American	26.2	11.6	2.9	5.1
Hispanic	36.0	18.0	5.6	10.4
**10th Graders**				
Total	49.8	27.2	13.7	14.7
Gender				
Boys	49.1	28.2	14.9	16.5
Girls	50.3	26.0	12.4	12.7
Race/Ethnicity				
White	52.1	29.1	15.6	16.1
African American	43.6	20.8	8.3	9.4
Hispanic	54.8	31.8	13.8	19.7
**12th Graders**				
Total	63.5	40.0	25.0	21.6
Gender				
Boys	63.3	42.1	27.5	25.5
Girls	63.5	37.5	22.0	17.6
Race/Ethnicity				
White	66.8	43.8	29.9	25.9
African American	55.2	30.1	14.2	11.3
Hispanic	65.3	39.7	20.0	20.8

* To derive percentages for each racial subgroup, data for the specified year and the previous year were combined to increase subgroup sample sizes and thus provide more stable estimates.

NOTE: For 8th graders, the approximate weighted *N* is 16,000. For 10th graders, the approximate weighted *N* is 14,900. For 12th graders, the approximate weighted *N* is 14,100.

SOURCE: The *Monitoring the Future study,* the University of Michigan.
